# Comparative Evaluation of Anti Microbial effects of Triple Antibiotic Paste and Amox and its derivatives against *E. Faecalis*: An *in vitro* study

**DOI:** 10.4317/jced.53053

**Published:** 2017-06-01

**Authors:** Manjeet Kaur, Shrikant Kendre, Parmod Gupta, Navneet Singh, Harsimran Sethi, Neha Gupta, Rushil Acharya

**Affiliations:** 1 Maharaja Ganga Singh Dental College and Research Centre 11LNT Hanumangarh Road, Shri Ganganagar, Rajasthan

## Abstract

**Background:**

*Enterococcus faecalis* is a microorganism commonly detected in asymptomatic, persistent endodontic infections. Triple antibiotic paste has stood the test of time as a proven antibiotic combination against *E. Faecalis*. However, problems with this include staining of teeth and standardization of the preparation. Thus, the search for better alternatives and better preparation techniques is still on.

**Aim:**

To observe the potential of combinations [(Amoxicillin+ Metronidazole, Amoxicillin Clavulanic Acid + Metronidazole; Amoxicillin and Cloxacillin + Metronidazole)] over Triple Antibiotic Paste.

**Material and Methods:**

Fifty single rooted teeth free from dental caries were selected for the study. Teeth were cut at equal distance from root apex (13mm from apex) with sterile diamond disk and straight hand piece for standardization of root length. The opening of root canal was enlarged with engine driven pro-taper files. To remove the organic and inorganic debris, canal was cleaned with 17% EDTA followed by 2.5% NaOCl for 5min. Distilled water irrigation was done for 5 min to remove any traces of used chemical and then sterilized in autoclave at 1200c for 15 min. at 15 lbs pressure. Bacteria cultured on blood agar plate and at the same time fresh antibiotic combinations were made and placed in the root canals, then incubated in the incubator, under sterile conditions and observed at 24hrs, 48hr and 72hrs.

**Results:**

The largest inhibition zones were observed for the Triple Antibiotic Paste, followed by Amoxicillin and Clavulanic acid + Metronidazole group however, the clearest zones were achieved with Amoxicillin and Clavlunic acid + Metronidazole group and the smallest for Amoxicillin and Metronidazole group.

**Conclusions:**

The results suggest that though Triple antibiotic showed the maximum inhibition, Amoxicillin and Clavulanic acid combination along with Metronidazole gave the most reliable results. Further studies using the different combinations and different concentrations along with different methods of increasing the shelf life of such medications can be undertaken.

** Key words:**Enterococcus faecalis, Triple Antibiotic Paste, Amoxcillin, Clavulanic acid.

## Introduction

Bacteria and their products play a key role in the initiation and perpetuation of pulpo-periapical pathosis. During root canal treatment Bacteria may be removed by filing or by chemical irrigation; but there is no concrete evidence in the literature that bio-mechanical preparation only, results in a bacteria-free root canal system (1). However, bacteria in the deeper layers of infected root dentin may occasionally remain and cause periapical impediment even after conventional root canal treatment (Ando & Hoshino1990) (2). Of these bacterial groups *Enterococcus faecalis* is most common bacteria present in teeth after the failure of root canal therapy. Its prevalence in such infections ranges from 24% to 77% (3,4).Studies have demonstrated that predictable disinfection of the root canal achieved only after proper antimicrobial medicaments are placement in the canals and left there in between appointments (5). The first reported local use of an antibiotic in endodontic treatment was in 1951 when Grossman used a poly-antibiotic paste known as PBSC (penicillin, bacitracin, streptomycin, and caprylate sodium) (1). Recently a number of antimicrobial agents were tried against *Enterococcus faecalis* but of these Triple Antibiotic Paste (TAP) has stood the test of time as a proven antibiotic combination against *E. Faecalis* (5-7) .

The combination of Metronidazole, Ciprofloxacin, and Minocycline (TAP) appears to be most promising and effective in penetrating dentinal tubules and sterilize root dentine (5). Yet, Problems with this include staining of teeth (8,9) and standardization of the preparation. Thus, the search for better alternatives and better preparation techniques is still on.

During day today practice Amoxicillin and its derivatives are often used as a first choice because of its broader spectrum of activity and bactericidal. Amoxicillin is absorbed more rapidly than and provides a higher and more continuous serum level. Augmentin is recommended as “fall-back” antibiotics when infections do not respond to traditional dental antibiotics (8,10).

The purpose of this study was to evaluate and compare the effect of amoxicillin and its commercially available derivates combination with Metronidazole and ciprofloxacin over triple antibiotic paste as an intra-canal medicament.

## Material and Methods

-Test organisms:

*Enterococcus faecalis* strain (ATCC 11700) was obtained from the Himedia Laboratories Pvt. Ltd., Mumbai, India. The bacterial cells were cultured in nutrient broth, harvested in incubator at a temperature of 370C for 24 hours. Pure cultures were then initially identified according to their Gram morphology and ability to produce catalase.

To sterile Blood Agar Base, which has been melted and cooled to 45 to 50°C, add 5% (vol/vol) sterile sheep blood that has been warmed to room temperature. Swirl the flask to mix thoroughly, avoiding the formation of bubbles, and dispense into sterile plates, continuing to avoid bubbles and froth on the surface. Follow it by incubation at 37°C for 24 hours.

Once blood agar plates were prepared, spreading of the bacteria from nutrient broth was done by using sterile cotton buds.

-Preparation of a specimen:

Fifty single-rooted teeth with complete root formation, which were free of dental caries, restorations and cracks, were selected. A low-speed diamond edge coated disc (Bredent®, Wittighausen, Senden, Germany) mounted on a milling machine under water cooling was used to section the teeth below the cementoenamel junction (12mm) to standardize the length of each specimen. Each root canal orifice was enlarged with an engine-driven enlarger sequentially (Gates Glidden drills 1, 2, 3) for easier application of the intracanal medicament. Biomechanical preparation was done using Rotary Protaper Files (Dentsply, Tulsa) (Sx, to F3) to standardize the internal diameter of canal.

The specimens were subjected to ultrasonic irrigation (EndoActivator, Dentsply, Weybridge, Surrey, UK) using 5.25% sodium hypochlorite (Clorox®, Oakland, California, USA) and then 17% EDTA (Calasept®, Nordiska Dental, Ängelholm, Skåne Country, Sweden) for one minute followed by normal saline rinse, in a sequence to remove organic and inorganic debris of the canal. As a sterilization protocol, the prepared specimens were autoclaved at 1210C for 30 min at 15 lbs pressure.

**Figure 1:**
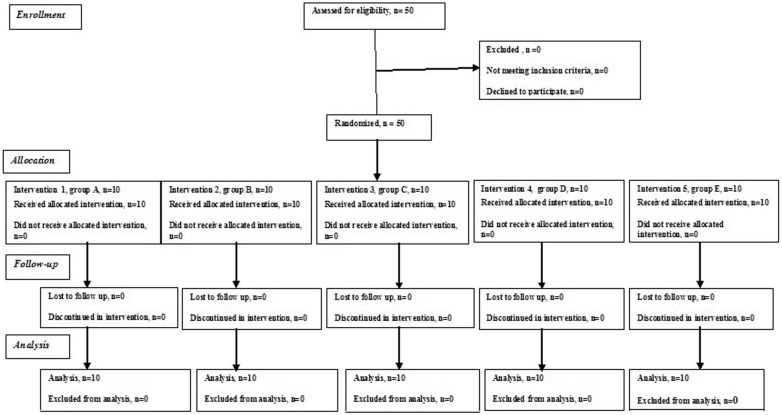
CONSORT flow diagram of the study process.

-Preparation of antibiotics:

Commercially available chemotherapeutic agents (Antibiotics) were selected and divided into groups. The 50 specimens were randomly divided into five groups (n=10), according to the intracanal medicament used (Fig. 1), as follows:

Group A- (Amoxicillin+ Clavulanic acid) 625 mg +Metronidazole 400mg.

Group B- Triple antibiotic paste.

Group C- (Amoxicillin + Cloxacillin) 500mg+Metronidazole 400mg.

Group D- Amoxicillin 500 mg + Metronidazole 400 mg.

Group E- Distilled water as matched untreated control group to provide base line data.

Individual antibiotics were triturated and mixed with 1ml of distilled water to formulate 1000:1 mg/ml concentration, and 0.1 ml of propylene glycol was added to each preparation to standardize the concentration of drug. From each preparation 0.5 mg was taken and mixed it to obtain paste like consistency before placement in prepared root canal.

Important note: Mixture of Amoxicillin and its combination with propylene glycol showed exothermic reaction and mix obtained was very granular and dry so, to overcome this problem distilled water was added to dissolve the antibiotic powder. Propylene glycol used because itself had antimicrobial activity and was used as it provides sustained release of drug for long duration of time.

-Placement of intracanal medicament:

Each specimen was taken individually and by using a #4 Lentulo spiral RA 25 mm (Dentsply Maillefer, Ballaigues, Switzerland) was selected by placing it passively up to 1 mm short of the working length before dressing the root canal with the medicament. Thereafter, the whole length of the Lentulo spiral was coated with the medicament paste and inserted up to 1 mm from working length. The procedure was repeated until the paste extruded from the coronal canal orifice indicating adequate fill.

To avoid any kind of apical extrusion, the apex was sealed using blue inlay wax. Following the placement of all intracanal medicaments inside the specimens, the coronal orifices were all sealed with Parafilm (Parafilm M®, Brand, Wertheim, Baden-Württemberg, Germany).

Bacteria were cultured on fifty blood agar plates and at the same time specimens were placed in each bacteria containing agar plate after removing inlay wax from apical region. The agar plates were placed in the incubator, under sterile conditions and in-hibition zones were observed after 24hrs, 48hr & 72hrs.

-Statistical analysis:

Data was normally distributed as tested by Tests of Normality Shapiro – Wilk test, ANOVA, Post-hoc Bonferroni test and paired t-test was used to assess difference between antibacterial efficacy between and within groups of antibiotics (*p*<0.05).

## Results

The means and standard deviations of the diameters of the growth inhibition zones for each concentration of the preparations are presented in  Table 1.

**Table 1:**
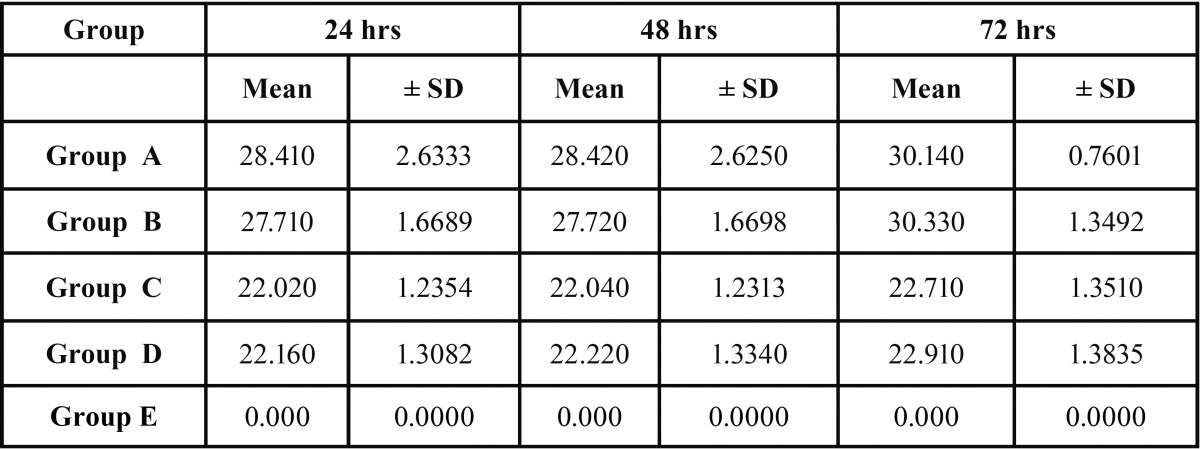
The means and standard deviations of the diameters of the growth inhibition zones for each concentration of the preparations.

After 24 and 48 hours Group A had the largest zones of growth inhibition followed by Group B, Group D, Group C. Group E showed no zone of inhibition ( Table 1). Whereas after 72 hours Group B had the largest zones of growth inhibition followed by Group A, Group D, Group C. Group E showed no zone of inhibition (T Table 1).

Overall, group A and group B had the largest zones of growth inhibition at all time intervals when compared to other groups. Group C and group D also had significant inhibitory effects on *E. Faecalis* at all time intervals compared to the control group (*P*<0.05).

**Figure 2:**
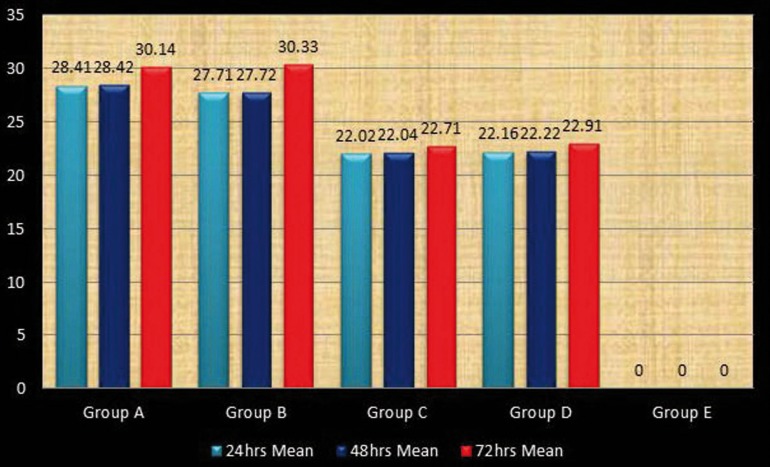
Multiple inter group comparison after 72 hour.

The Inter group comparison after 72 hours revealed no statistically significant differences when Group A was compared with Group B and when Group C was compared with Group D (*p* =1.000). When Group A was compared with Group C, D and E, statistically significant differences (*p* < 0.001) were obtained. Similar results were obtained when Group B was compared with Group C, D and E (*p* < 0.001) ( Table 2, Fig. 2).

**Table 2:**
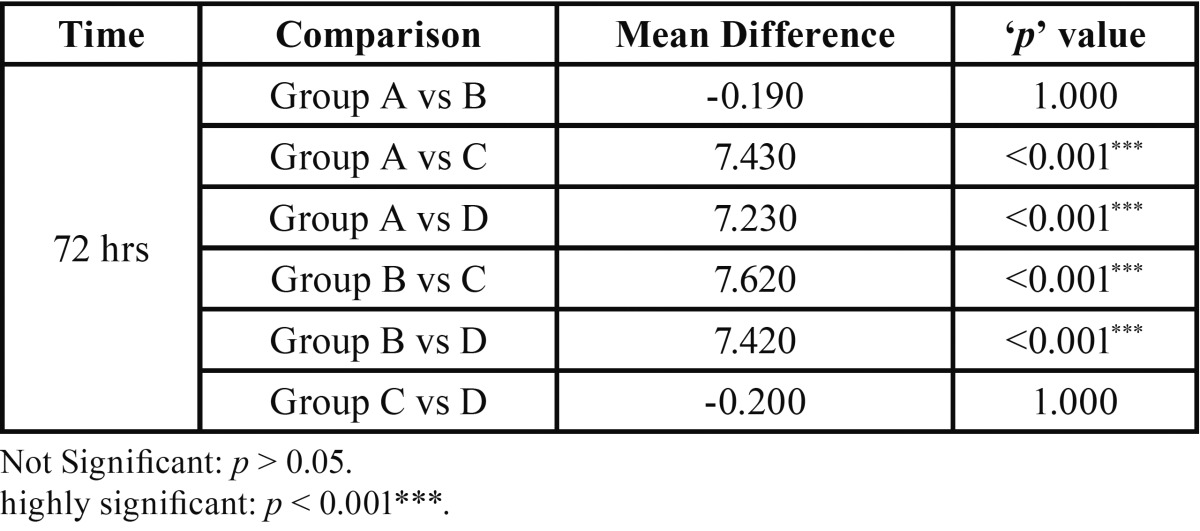
Multiple inter group comparison after 72 hour.

## Discussion

Modern concept of medicine emphasizes prevention and reversal of the diseases. Only when these attempts fail, one would take on the another unfavorable approaches, i.e., surgical intervention and restoration with artificial prostheses (5,11). The successful treatment of both primary and secondary endodontic infections involves effective eradication of causative micro-organisms during the root canal treatment procedures by disrupting and destroying the microbial ecosystem through chemical and mechanical methods (12).

 Numerous measures have been described to reduce the number of micro-organisms using instrumentation techniques, irrigation regimens, and intracanal medicaments. According to Soares, chemo-mechanical instrumentation promotes a partial and temporary antisepsis, regardless of the instrumentation technique and concentration of the irrigating solutions (13). Peters LB *et al.*, Orstavik D *et al.* observed that chemo-mechanical preparation is often not enough, and many bacteria may remain in the root canal system (14,15).

Studies have reported that inter-appointment intracanal medications have been undeniably shown contribution to favorable outcomes when treating endodontic infection by eliminating or destroying any remaining viable bacteria in the root canal system that have not been destroyed by the chemo-mechanical preparation processes (i.e., instrumentation and irrigation) (13-16).

According to Nakornchai *et al.*, the 3-Mix antibiotic paste containing Ciprofloxacin, Minocycline and Metronidazole is superior to vitapex for root canal treatment of pulpally involved primary molars (17). But Kim *et al.*, and Lenherr *et al.*, identified the discoloration caused by Minocycline used in tri-antibiotic paste (18,19). Thomson and Kahler substituted Amoxicillin for Minocycline in their case report to avoid this discoloration (20). Hence in the present study a new combination of antibiotics was used containing commercially available Amoxicillin and its derivatives, Metronidazole and Ciprofloxacin to compare effectiveness against TAP.

Selection of the antimicrobial agents in present study was based on prior studies that tested the susceptibility / resistance patterns of *E. Faecalis*. Penicillin and Ampicillin were not selected because exclusive strains of *E. Faecalis* express the B-lactamase enzyme; instead, amoxicillin (a selective inhibitor of bacterial cell wall synthesis) with Clavulanic acid (a β-lactamase inhibitor) and Cloxacillin was used (8).

The ideal or optimum vehicle for delivery of antibiotics in root canal should have ability to facilitate better diffusion of medicament through dentinal tubules and anatomical aberrations like fins, isthmuses and blocked canals. Therefore diffusion of antibiotic into cementum and periradicular tissue may be advantageous. Hoshino *et al.* used propylene glycol and macrogol for delivery of triple antibiotic paste (21). Cruz EV *et al.* investigated the penetration effect of propylene glycol into root dentine. The area and the depth of penetration of Safranin O dye in propylene glycol were reported to be significantly greater than dye with distilled water into root dentine (22). The presence of smear layer significantly delayed the penetration of the dye in this study indicating the need for their removal for better diffusion of medicament (2). Thus Propylene gycol is a useful vehicle for delivering intracanal medicaments in root canal system.

In present study Intra-group analysis indicated that all the medicaments succeeded in promoting reduction in bacterial growth which was calculated by measuring zone of inhibition. Group B (TAP group) was significantly more effective at killing *E. Faecalis* followed by group A as compared to other groups. But there was not significant found when group A was compared with B (*p*=0.05). This result did not come as a surprise because all the agents used were bactericidal agents. Earlier it has been shown that the triple antibiotic paste and Amoxicillin+ Clavlunic acid combination was the most effective in sterilization of root canal dentin (8).

In order to obtain the maximum clinical benefit of the anti-bacterial agents used in this mode, additional research should be carried out to investigate the best drug delivery form, drug substantivity and the feasibility of using drug combinations. This would be more practical if the biofilm model was a polymicrobial one. Also possible side effects in the form of sensitization, development of resistant strains or any alteration of root canal dentin surface characteristics should be investigated.
